# A unified deep-learning network to accurately segment insulin granules of different animal models imaged under different electron microscopy methodologies

**DOI:** 10.1007/s13238-018-0575-y

**Published:** 2018-10-10

**Authors:** Xiaoya Zhang, Xiaohong Peng, Chengsheng Han, Wenzhen Zhu, Lisi Wei, Yulin Zhang, Yi Wang, Xiuqin Zhang, Hao Tang, Jianshe Zhang, Xiaojun Xu, Fengping Feng, Yanhong Xue, Erlin Yao, Guangming Tan, Tao Xu, Liangyi Chen

**Affiliations:** 1 State Key Laboratory of Membrane Biology, Beijing Key Laboratory of Cardiometabolic Molecular Medicine, Institute of Molecular Medicine, Peking University, Beijing, 100871 China; 2 National Laboratory of Biomacromolecules, CAS Center for Excellence in Biomacromolecules, Institute of Biophysics, Chinese Academy of Sciences, Beijing, 100101 China; 3 University of Chinese Academy of Sciences, Beijing, 100049 China; 4 Drug Discovery Center, Key Laboratory of Chemical Genomics, Peking University Shenzhen Graduate School, Shenzhen, 518055 China; 5 Marine Science College of Zhejiang Ocean University, National Engineering Research Center of Marine Facilities Aquaculture, Zhoushan, 316022 China

**Dear Editor,**

Insulin is important for body metabolism regulation and glucose homeostasis, and its dysregulation often leads to metabolic syndrome (MS) and diabetes. Insulin is normally stored in large dense-core vesicles (LDCVs) in pancreatic beta cells, and significant reductions in the number, size, gray level and density of insulin granules confer diabetes both in mice (Xue et al., 2012) and humans (Masini et al., 2012). Due to the difficulty of obtaining human islet samples, many works use mice as the animal model. However, the architecture of normal islets in humans differs significantly from that of rodents (Cabrera et al., 2006). Beta cells in the mouse islet core are surrounded by the mantle comprising of alpha and delta cells, whereas alpha, beta and delta cells are intermingled in human islets. The structural differences suggest a possible difference in islet function. In this sense, non-human primates such as rhesus monkeys are a better model, as their islets share a similar architecture with humans (Cabrera et al., 2006). The quantitative nature of the insulin granules within monkey islet beta cells, and whether they change during metabolic dysregulation remain to be explored. Under the electron microscope (EM), insulin granules are usually spherical organelles containing an electron dense-core separated from the surrounding membrane by a halo, with a size of ranged from 100–800 nm (MacDonald et al., 2006) in mouse beta cells. This number is estimated as ~10,000 per beta cell. Because thin-section EM do not necessarily provide the correct spatial coordination of granules within one beta cell, recent years have witnessed the emergence of volumetric electron microscopy techniques such as electron tomography and focused ion beam scanning electron microscopy (FIB-SEM) (Briggman and Bock, 2012).

For the first time, we collected three-dimensional images of pancreatic beta cells in wild type (WT) and MS rhesus monkeys with a FIB-SEM and manually annotated granules from a relatively small number of images. Because the morphological and structural natures of insulin granules are important for their optimal function, quantitative and automatic analysis of insulin granules in islets is important. Manually segmenting densely distributed LDCVs is a labor-intensive task due to the big datasets brought by saturated and continuous sampling in the lateral and axial axes. Although several semi-automated segmentation methods for rodent LDCVs have been proposed (Diaz et al., 2010), they are built on time-consuming and human-designed features that cannot adapt to micrographs of different magnification and are extremely prone to errors for images with low signal-to-noise ratios. Nevertheless, the machine learning field has witnessed a flourishing of “deep-learning” algorithms. Since AlexNet outperformed all other algorithms by a large margin in the ImageNet contest in 2012, a variety of deep-learning methods for image segmentation have been widely used, including the standard convolutional networks (CNN) (Van Valen et al., 2016), and fully convolutional networks (FCN) (Long, 2014). Recently, multi-scale features, dilated convolutions, context encoding, conditional random fields (CRFs) are incorporated to FCNs to improve spatial resolution, bringing more novel and complicated network structures such as Tiramisu (Jegou et al., 2017), Deeplab (Chen et al., 2018). However, different from natural images, we only have a small electron micrograph dataset annotated, in which insulin granules only occupy a small portion of the image. In order to prevent overfitting, we take concise deep-learning networks as the starting point, such as U-Net (Ronneberger et al., 2015).

To automatically and precisely segment the insulin granules with different sizes and pattern features in EM images, we built and trained a multi-branch fully convolutional network (MFCN), which consists of three modules: a multi-scale inception module, a multi-branch sampling module, and a multi-scale ensemble module (Fig. 1). The “multi-scale” design was inspired by the naïve inception module (Szegedy et al., 2015), which uses different sizes kernels to extract both coarse and fine grained features. Although some papers have proposed that a stack of two 3 × 3 convolutional layers has an effective receptive field of 5 × 5, our experiments show that larger kernels may be more robust in extracting features and less prone be noisy in EM images (Table S1). Next, the extracted features are linked in tandem and in parallel to form the multi-branch module. Specifically, we have combined three hierarchical convolutional encoding-decoding branches (blue, green and light red) with different rates of down-sampling and up-sampling (blue: 16×; green: 8×; light red: 4×). Thus three branches act as three binary classifiers with different receptive fields, ended with two score maps that are sliced and then concatenated by 1 × 1 convolutional layer for feature weighting (the multi-scale ensemble module). The outputs are transformed by the softmax layer in the end to provide the classification probability for the final decision.

**Figure 1:**
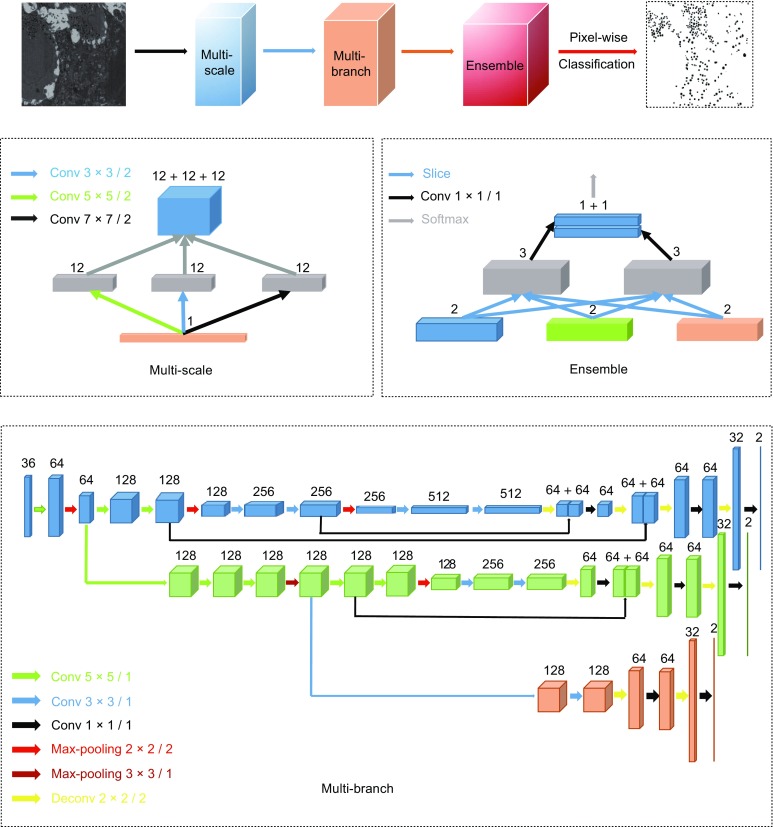
**Architecture of MFCN**. The MFCN includes three modules: a multi-scale inception module, a multi-branch sampling module and a multi-scale ensemble module. The multi-scale module uses three different kernel size convolutional layers (3 × 3, 5 × 5, 7 × 7, stride 2) to extract features, which are expanded to form branches concatenated with other modules, respectively. In multi-branch module, we have combined three branches (blue, green and light red) with different steps of down-sampling and up-sampling (blue: 16× down-sampling and up-sampling; green: 8× down-sampling and up-sampling; light red: 4× down-sampling and up-sampling). Finally, the ensemble module combines multi-mode features extracted from the multi-branch module to detect vesicles with different sizes and shapes. The number of channels of feature maps is denoted on top of the cuboids and the arrows denote the different operations (kernel size/stride).

The whole image processing can be divided into three parts (Fig. S1). First, we used histogram equalization for image pre-processing, which homogenized the uneven illumination (Fig. S2), and proved to significantly speed up the convergence of the network. Next, we fed the MFCN network with pre-processed images for the binary segmentation maps. Finally, we used a simple watershed based edge detection method for the instance segmentation of the binary maps. Based on the results of the final step, we could quantitatively extract spatio-temporal information for each granule, such as the coordinates of the boundary, areas, mean gray level values and perimeters.

Side-by-side, we compared the performance of our method with other previously published methods, including the random forest (Smith and Frank, 2016), the standard convolutional networks (Van Valen et al., 2016), and the U-Net (Ronneberger, 2015). For an objective and fair comparison, we adopted two sets of evaluation metrics. One was used for measuring classification accuracy of each pixel, including pixel accuracy, mean accuracy and mean region intersection over union (mean IU) (Long, 2014). The other was used for evaluating each segmented granule, including true positive (TP) false positive (FP), true negative (TN) and false negative (FN) (detailed in Supplementary Materials).

From Figures. S3 and S4, we could infer that the random forest algorithm performed the worst, despite the high pixel accuracy it achieved (96%, Table S2). This discrepancy was because the insulin granules occupied only a small part of the whole EM image. The standard CNN network outperformed the random forest algorithm in several aspects but their results were variable among different images (Table S3). As it only perceives local semantic information from fixed and small size image patches, the standard CNN may not be suitable for detecting granules of different sizes and shapes. In addition, it needed more time than other methods, as it was computationally intense to calculate many redundant, overlapping patches for segmentation. U-Net, a typical variant of FCN for biomedical segmentation, is characterized by a U-shaped architecture containing symmetrical down-sampling and up-sampling blocks. Better than the random forest and CNN algorithm, it still achieved only ~68% precision in detecting insulin granules from WT and MS islets (Table S3). Relatively, MFCN reached ~87% precision in detecting granules (Table S3). Besides, in detecting insulin granules of low signal-to-noise contrast, U-Net segmented granules were more irregular, non-continuous and fragmented than MFCN (Figs. S3 and S4). Apart from that, many dark regions within the nucleus were incorrectly detected as granules by U-Net but not the MFCN, which led to high error rate of the former (Fig. S5).

Compared with U-Net, MFCN trimmed off several redundant skip-layer connections, added multi-branch down-sampling, and combined multi-scale contextures to produce the final output. Benefiting from these features and the combination of receptive fields of different sizes, we have demonstrated the robustness of MFCN in detecting granules with diversified sizes and shapes while rejecting dark non-granular structures, which is superior to other algorithms tested (Tables S2 and S3). Having established the robustness and superiority of the current configuration of MFCN, we tested whether the trained network could be used to segment insulin granules of different species and EM images using different microscopes. We used the same MFCN network to detect insulin granules isolated from mouse islets and imaged under the STEM tomography and thin-slice TEM (Fig. S6). Without any fine-tuning, our network, trained on the FIB-SEM dataset, readily resolved insulin granules of different signal-to-noise ratios in the tomography data and the granules of various intensities from single-slice TEM data. As a result, the trained MFCN is insensitive to image resolution, light intensity, type of electron microscope, and animal species, and thus confers robustness and transferability.

We quantitatively analyzed the structures of insulin granules from beta cells isolated from WT and MS rhesus monkeys. As representative examples shown in Fig. 2A and 2B, insulin granules in the beta cells of spontaneous MS differed from those of WT monkey. The density of granules increased nearly 2-fold in the beta cells of MS monkey compared with WT monkey (2.3 ± 0.19 vs. 1.03 ± 0.11 per μm^2^, Fig. 2C), indicating the possible adaptation of hyperinsulinemia to enhanced insulin resistance, which is also found in the pathology of type II diabetes in mice and humans. On the other hand, the size of dense-cores and insulin granules in MS beta cells decreased compared with the control (Fig. 2D and 2E). Moreover, the shape of the granule dense-core also became irregular (Fig. 2F), and some dense-cores exhibited rod-like shapes (indicated by arrowhead in Fig. 2B), similar to those reported in the ZnT8 KO mouse (Wijesekara et al., 2010). Therefore, despite an increase in the number of insulin granules in the MS monkey, the changes in the size and shape of granule cores may represent early dysregulation of insulin granule biogenesis. We also analyzed the spatial distributions of insulin granules in WT and MS beta-cells (Fig. 2G–I). Along with increase in insulin granules in MS beta-cells, there was absolute increase in numbers of granules that resided ~100 and ~300 nm within the plasma membrane. However, by analyzing the relative frequency of granules distribution within 100 nm regions close to the plasma membrane, we found that WT beta-cells exhibited more enrichment of granules that were less than 40 nm to the plasma membrane as compared with the MS ones, indicative of defective docked granules in MS beta-cells.

**Figure 2:**
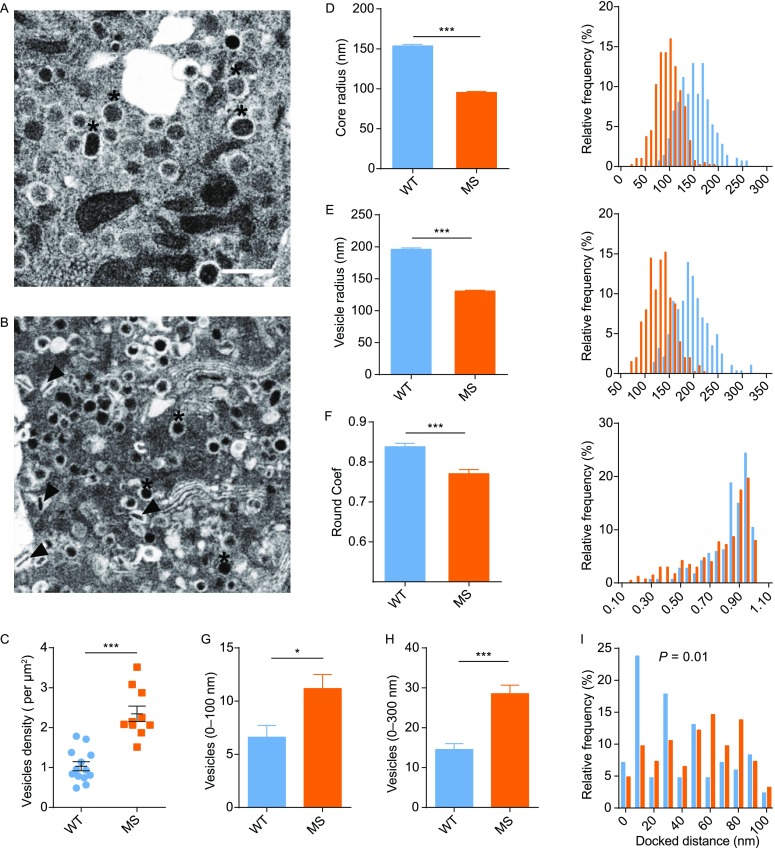
**Quantitative comparison of insulin granules from beta cells of WT and spontaneous MS rhesus monkeys**. (A and B) Representative EM images of WT (A) and MS (B) beta cells. Typical normal, symmetric and round granules were labeled by asterisks, and whole granules of abnormal shapes were indicated by arrowhead. Scale bar, 1 μm. (C) Density of insulin granules (*n* = 958) in MS monkey beta cells (*n* = 10) is significantly higher than that (*n* = 627) in WT cells (*n* = 13) (*P* < 0.001 by Student’s *t*-test). (D) Average (left) and distribution (right) of radius of dense-cores, in which MS beta cells displayed a significant left-shift distribution (*P* < 0.0001 by Kolmogorov-Smirnov test). (E) Average (left) and distribution (right) of radius of insulin granules, in which MS beta cells again displayed a significant left-shift distribution (*P* < 0.0001 by Kolmogorov-Smirnov test). (F) Average (left) and distribution (right) of round coefficient of insulin granules, in which MS beta cells exhibited an irregular shape (*P* < 0.0001 by Kolmogorov-Smirnov test). (G and H) In MS beta-cells (*n* = 11), there were absolute increase in numbers of granules that resided ~100 (G) and ~300 nm (H) within the plasma membrane compared with WT ones (*n* = 13). (I) WT beta-cells exhibited more enrichment of granules that were less than 40 nm to the plasma membrane as compared with the MS ones. (*P* = 0.01 by Kolmogorov-Smirnov test).

In summary, we have developed a novel deep learning framework to auto-segment insulin granules from EM images from WT and MS rhesus monkey beta cells. We show that the proposed MFCN has outperformed other algorithms in resolving insulin granules of distinct shapes and sizes, and offers good transferability in handling of data from different electron microscopes. Therefore, MFCN can be represented as a significant step toward fully automated segmentation and quantification of insulin granules from EM images. We believe that the MFCN and its underlying principles could be used for other classification problems in biological or medical image analysis in general. Applying this network to analyze the morphology and spatial distributions of insulin granules in beta cells of MS rhesus monkeys has already provided some insights. First, we confirmed that the morphology of insulin granules in rhesus monkeys is similar to that of humans or rodents. Second, as the number of granules per unit area of cytoplasm increased in MS monkeys compared with the control, this possibly reflects a compensatory increase in insulin synthesis at the early stage of diabetes in non-human primates as well. Third, the sizes and shapes of dense-cores changed in the beta cells of MS monkeys, as there were more empty granules or rod-like dense-core granules in the diseased animal. As the dense-core is produced by the co-crystallization of Zinc and insulin, these changes suggest that defects in insulin synthesis, packaging or crystallization may manifest at the early stage of disease development when there is an absolute increase in insulin granules. Finally, the defective docked granules found in MS beta-cells are consistent with the down-regulation of SNARE proteins and defective docking of insulin granules in the beta-cells from diabetic rodents and patients (Ostenson et al., 2006). All these findings may help to address this theory and prove insights into the diabetes progression process in humans.

## Electronic supplementary material

Below is the link to the electronic supplementary material.

Supplementary material 1 (PDF 2449 kb)
