# Supplementary material for: A unified deep-learning network to accurately segment insulin granules of different animal models imaged under different electron microscopy methodologies
Source: Protein Cell. 2018 Oct 10;10(4):306–11. doi: 10.1007/s13238-018-0575-y (PMC6418072; doi:10.1007/s13238-018-0575-y)
Supplement: Supplementary file 1 — Supplementary material 1 (PDF 2449 kb) [file 13238_2018_575_MOESM1_ESM.pdf]

SUPPLEMENTARY MATERIALS

Dataset introduction

We established a small, manually segmented dataset, including 27 FIB-SEM images from WT monkey islets and 67 images from the MS monkey islets, each with the resolution of 1792×2048.

The annotated image sequence (saved as *data.tiff*) is a set of 94 sections that are randomly selected from different groups of focused FIB-SEM datasets for the insulin granules from WT and MS rhesus monkeys.

The corresponding labels (saved as *label.tiff*) for binary segmentation are provided in an in-out fashion, i.e., black for the pixels of vesicles and white for the other pixels (which correspond to other structures within beta-cells or alpha-cells, such as the nucleus and cytoplasm).

Optimization of the MFCN

Suppose a training dataset $\mathrm{S}=\{(\mathrm{x}^n, y^n), n=1,...,m\}$, containing *m* images, each composed of L pixels. (x^n, y^n) denoted n th input image and its segmented ground truth respectively. For a multi-class problem with *k* classes in total, we define $f(\theta, x)$ as the input of the softmax layer (θ represents parameter of the whole net). For each pixel l in an image pair $(x^{(i)}, y^{(i)})$, we defined the predicted probability of it belonging to class j ($j \in N$) to be

$$p\{\tilde{\mathrm{y}}_l^{(i)} = j\} = \frac{e^{f(\theta; x^{(i)})_{l,j}}}{\sum_{j=0}^{k-1} e^{f(\theta; x^{(i)})_{l,j}}}, \tag{1}$$

and the corresponding cost function at each pixel l as :

$$J_l(\theta; x, y)^{(i)} = -\sum_{j=0}^{k-1} 1\{y_l^{(i)} = j\} \log p\{\tilde{y}_l^{(i)} = j\} \tag{2}$$

Specifically, since discriminating the vesicle from the non-vesicle region is a binary classification, $y_l^{(i)} \in \{0,1\}$, we defined:

$$h_\theta(x) = \frac{1}{1 + \exp(-f(\theta; x))}, \tag{3}$$

then the cost function $J_l(\theta)$ can be rewritten as:

$$J_l(\theta; x, y)^{(i)} = -y_l^{(i)} \log h_\theta(x^{(i)})_l - (1 - y_l^{(i)}) \log(1 - h_\theta(x^{(i)})_l) \tag{4}$$

Finally, we added a regularization item to the model to avoid the overfitting problem, and obtained the final cost function as:

$$J(\theta) = [\frac{1}{m} \sum_{i=1}^{m} \sum_{l=1}^{L} J_l(\theta; x, y)^{(i)}] + \frac{\lambda}{2} \|\theta\|^2 \tag{5}$$

The latter part of equation (5) is $L2$ regular term in order to prevent overfitting, improve the generalization of the model, and help to solve the matrix with bad condition number. We selected $L2$ regularization with a small λ of 0.0001 in experiments.

Our goal is to minimize the $J(\theta)$ as a function of the model parameters θ. In model learning, parameters are updated by backpropagation and we follow the optimization process of the classic stochastic gradient descent with momentum to train the model. Specifically, by combining the gradient of the whole cost function $\nabla J(\theta)$ and the current velocity vector V_t, we update the parameters θ as follows:

$$V_{t+1} = \mu V_t - \alpha \nabla J(\theta) \tag{6}$$

$$\theta_{t+1} = \theta_t + V_{t+1} \tag{7}$$

where α represents the learning rate, and μ is the momentum coefficient.

In the training process, we used a combination of Gaussian distribution and random

numbers from the Xavier algorithm to generate the initial values of the model parameters.

Segmentation of new images

Suppose a testing dataset *T* with *N* images is $T = \{t^1, t^2 ... t^N\}, t^i \in R^{m*n}, m = 1792, n = 2048$, for a pixel *l* in an test image *i*, its predicted result $j_l^{(i)*}$ can be expressed as:

$$j_l^{(i)*} = \arg\max \frac{e^{f(\theta; t^i)_{l,j}}}{\sum_{j=0}^{1} e^{f(\theta_j; t^i)_{l,j}}} \quad , \quad j_l^{(i)*} \in \{0,1\} \qquad (8)$$

We found other post-processing procedures such as CRF (Chen, 2016) rendered the segmented grey granules smaller and more non-continuously, so this was not used here.

Experimental settings

To reduce the dependence of the trained network on only specific proportion of the dataset and the network performance deviations, we used 5-fold cross-validation. By randomly dividing the dataset into five equal parts, we selected four for model training, and used the remaining group for model testing. We performed the same cross-validation procedure five times and used the average results. In the training stage, we randomly cropped each image to 640×704 pixels to reduce memory consumption and training time, while keeping the size of images for testing or validation unchanged. Random cropping could also be used for data augmentation to prevent overfitting. The pre-processing and post-processing were implemented by Matlab, and the training and testing of MFCN were under the open-source deep learning framework Caffe library (Jia, 2014) with a Tesla K40M GPU. All of the software and our annotated dataset described here, is available at *https://github.com/644zhang/MFCN.*

Implementation of various segmentation methods

we compared the performance of our MFCN method with other previously published methods. First, following a previous report (Diaz et al., 2010), we implemented a conventional rule-based segmentation that involved the top-hat, the watershed transformation and the Hough transformation. However, this method only detected insulin granules of high contrast and most with regular shape, thus performed poorly when came to our problem of granule segmentation. Thus, we removed this method from the comparison tables and limited the comparisons only among different machine learning algorithms.

We implemented random forest algorithm embedded in WEKA (Smith and Frank, 2016), which is a classical machine learning method in a wide-used platform. Here we selected the default training features including Gaussian blur, Sobel filter, Hessian, Difference of Gaussians, and Membrane projections, and used 200 decision trees for the training. The second one was the standard convolutional networks (Van Valen et al., 2016), in which we selected a sliding window of 61×61 pixels to approximate the size of granules in our data for patch-wise prediction. In these two methods, we selected the most suitable procedures and parameters. The last one was a revision of U-Net (Ronneberger, 2015), in which we followed the same procedure to train the network as that used for MFCN. A different point is that we fine-tuned the U-Net based on the already trained network (Ronneberger, 2015) to ensure the U-Net starting from a good initialization point, while trained the MFCN from scratch when the optimization became harder.

Evaluation metrics

We adopted two sets of evaluation metrics. One was designed for the common semantic segmentation and scene parsing (Long, 2014) , including pixel accuracy, mean accuracy, and mean region intersection over union (Mean IU), as defined by the following equations:

$$\text{Pixel Accuracy: } \sum_{\mathrm{i}} n_{ii} / \sum_{i} t_i \text{ ;}$$

$$\text{Mean Accuracy: } (1/n_{cl})\sum_i n_{ii}/t_i;$$

$$\text{Mean IU: } (1/n_{cl})/\sum_i n_{ii}(t_i+\sum_j n_{ji}-n_{ii});$$

in which n_{ij} quantifies the number of pixels in class *i* predicted to be in class *j*, and $t_i=\sum_j n_{ij}$ denotes the number of pixels belonging to class *i*, with n_{cl} classes in all.

However, the metrics based on the single pixel may not be able to exactly and correctly evaluate the segmentation accuracy of insulin granules. Thus, we also designed a metric based on detecting single objectives such as granules. In segmenting the single granules, we regarded an intersection over union (IoU) between the manually annotated region and the prediction region larger than 0.5 as a correct prediction. The number of correct or wrong predictions was used to calculate five ratios as follows. Specifically, we defined TP (True Positive) as correctly identified granules, FP (False Positive) as incorrectly identified granules, TN (True Negative) as correctly rejected non-granular structures, and FN (False Negative) as incorrectly rejected granular structures. With these, we calculated the following parameters:

Precision Ratio = TP / (TP + FP)

Recall Ratio = TP / (TP + FN)

Error Ratio = (FP + FN) / (TP + FN)

Miss Ratio = TN / (TP + FN)

F-measure = 2 × P × R / (P + R)

Experiments on different structures of MFCN

We have tried to benchmark the performance of proposed multi-scale inception and multi-branch modules against MFCNs with different structures, such as replacing the multi-scale inception module with one fixed size of kernel, replacing three branches with one branch, four branches, or five branches modules, etc. From **Table S1**, we concluded that models of one branch or of branches more than 3 did lead to reduced

mean accuracy and mean IU. If the inception module was replaced with small kernels like 3×3, we also found a degenerated performance of the model. From these experiments, we conclude that the current network design is the most suitable for the segmentation of insulin granules in EM images.

Islet isolation and culture from rhesus monkeys

Dissected pancreases from rhesus monkeys (*Macaca mulatta*, aged 14-19 years) were obtained from the Non-human Primate Research Center, Peking University, Beijing, China (Zhang et al., 2011). The study was approved by the Ethics Committee of Peking University and performed in the animal facility of Peking University accredited by the Association for Assessment and Accreditation of Laboratory Animal Care International. Procured pancreases were stored in Belzer-UW solution and briefly sterilized with Betadine (5 mg/ml), Fungizone (2.5 mg/ml) and Cefazolin (0.5 mg/ml), sequentially. The islets were isolated by enzymatic digestion (0.5 mg/ml collagenase Type 1 and collagenase P) for 30 min at 37℃ and hand-picked. Isolated islets were cultured in M1066 medium containing 10% FBS and 5.5 mmol/l glucose in a 5% CO_2 incubator for 12~24 hours.

FIB-SEM

Islets were harvested and fixed in 2.5% glutaraldehyde in PBS with a pH of 7.4 at room temperature for 1 hour. Samples were post-fixed with 1% osmium tetroxide in 0.1 mol/L sodium cacodylate for 1.5 h and stained with 2% (w/v) uranyl acetate in double-distilled water for 50 min to increase the contrast under SEM. After washing and dehydration in a graded series of acetone, samples were embedded in Embed 812 resin.

The resin-embedded block was trimmed with a diamond knife using a Leica ultra-microtome EM UC6 (Leica, Germany). The block was remounted with the exposed islet upwards and glued for use. The surface of the block was coated with a thin layer of carbon to increase the electrical conductivity. The block was transferred into the FIB-SEM chamber, and sectioned with a Helios Nanolab 600i dual-beam

SEM (FEI, the Netherlands), which combined high-resolution field-emission SEM with a focused gallium ion beam. A 1-mm-thick layer of platinum was deposited on the block above the region of interest to protect the specimen and reduce FIB milling artifacts. Automated sequential FIB milling and SEM imaging were conducted as described previously (Merchan-Perez et al., 2009). A layer of platinum (~0.8 mm thick) was deposited on a surface perpendicular to the block face. The block face was imaged using an electron beam with an acceleration voltage of 3 kV, a current of 0.17 nA and a dwell time of 10 μs. The image was captured with a 2K×2K CCD camera with a horizon field width (HFW) at 13.8 μm. After the surface was imaged, a gallium ion beam with an acceleration voltage of 30 kV and a current of 0.79 nA was used to remove a 20-nm thick superficial layer from the block face for the next round of imaging and milling.

Statistic analysis

To benchmark MFCN with other algorithms, we randomly selected electron micrographs to be segmented. Morphological parameters of individual insulin granules were assessed and quantified using various algorithms in combination with manual processing in ImageJ (NIH, USA). All results were presented as a frequency distribution histogram or mean ± SEM. Statistical significance was evaluated by Student *t*-test or Kolmogorov-Smirnov test using GraphPad Prism 6. Asterisks denoted statistical significance compared with the WT, with p values less than 0.05(*), 0.01(**), and 0.001(***).

Supplementary Figures and Legends

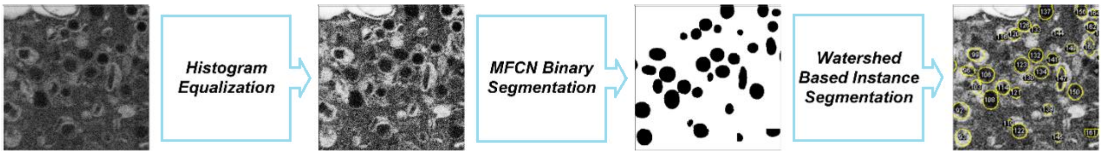

Figure S1. Flow diagram of whole image processing procedure. Whole image processing can be divided into three parts: histogram equalization, MFCN binary segmentation, and watershed-based instance segmentation.

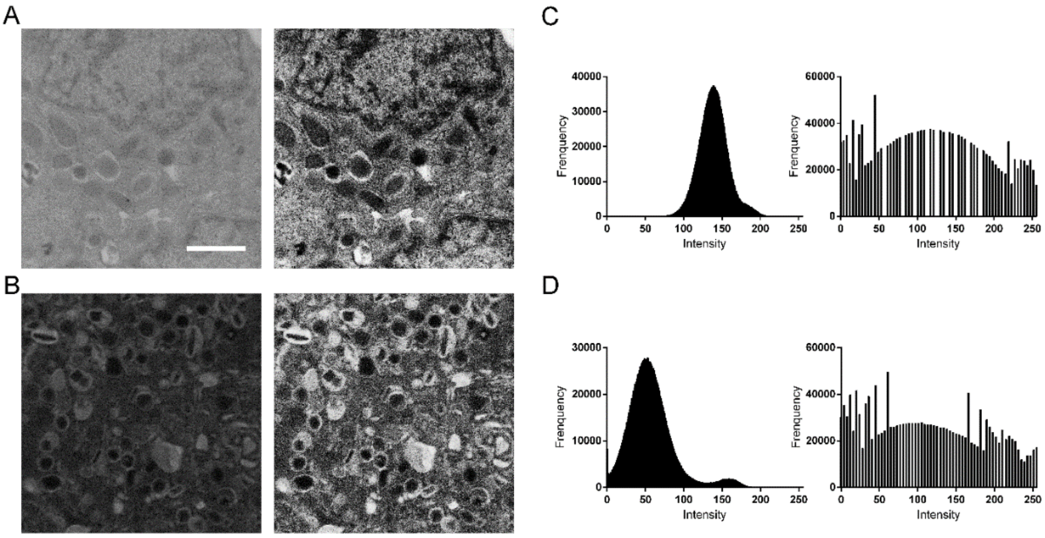

Figure S2. Histogram equalization for efficient image pre-processing. (**A, B**) FIB-SEM images of beta cells from WT (**A**) and MS (**B**) monkeys in original format (left) and after processing with histogram equalization (right). (**C, D**) Intensity histograms of images in (**A**, **B**) before (left) and after (right) histogram equalization. Scale bar, 1 µm.

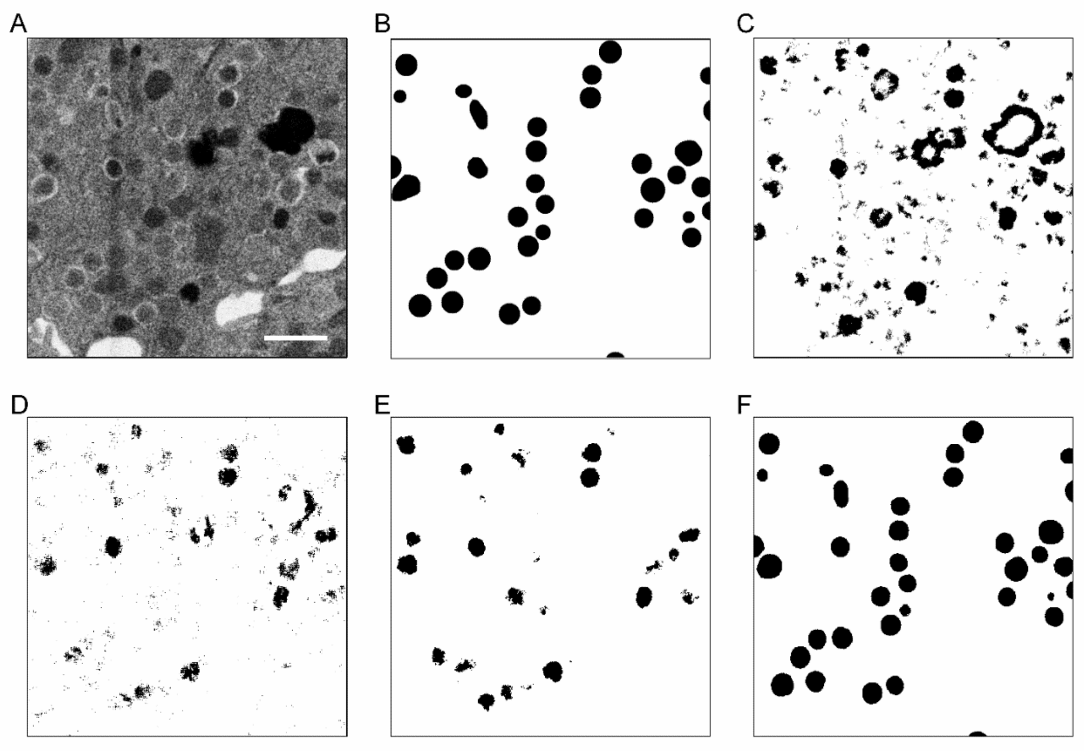

Figure S3. Binary segmentation maps of insulin granules in WT monkey beta cells by four algorithms. (**A**) Original image. (**B**) Manually labeled segmentation as gold standard. (**C**) Segmentation result of random forest algorithm. (**D**) Segmentation result of standard CNN algorithm. (**E**) Segmentation result of U-Net algorithm. (**F**) Segmentation result of MFCN algorithm. Scale bar, 1 μm.

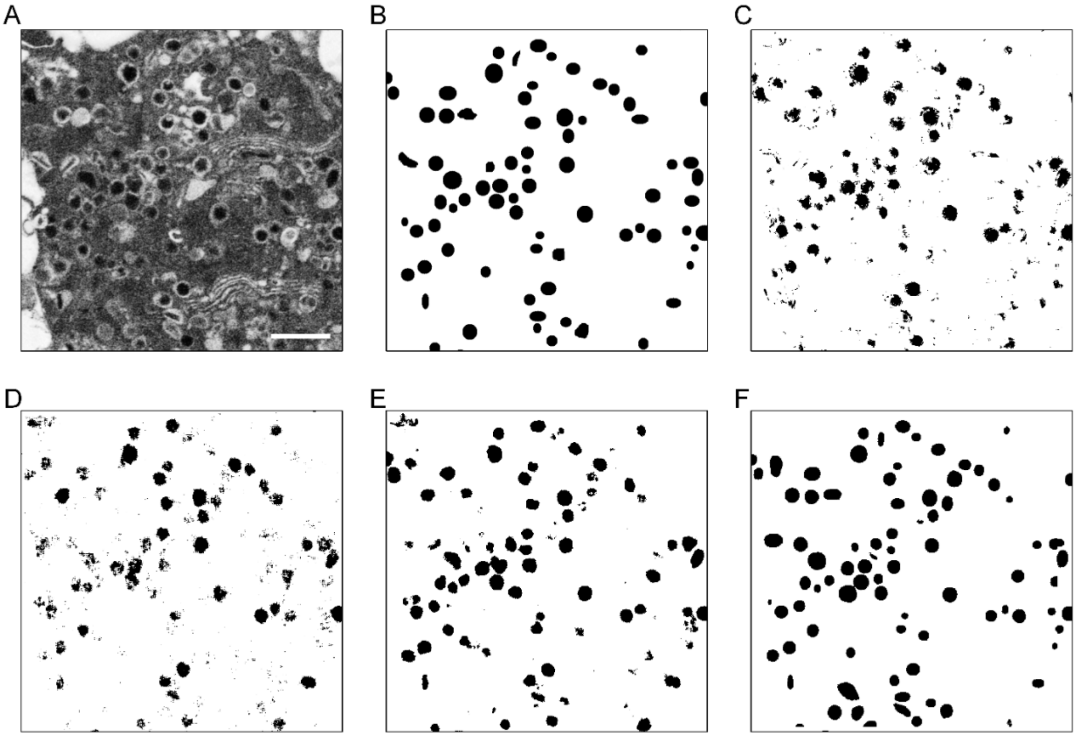

Figure S4. Binary segmentation maps of insulin granules in MS monkey beta cells with four algorithms. (**A**) Original image. (**B**) Manually labeled segmentation as gold standard. (**C**) Segmentation result of random forest algorithm. (**D**) Segmentation result of standard CNN algorithm. (**E**) Segmentation result of U-Net algorithm. (**F**) Segmentation result of MFCN algorithm. Scale bar, 1 μm.

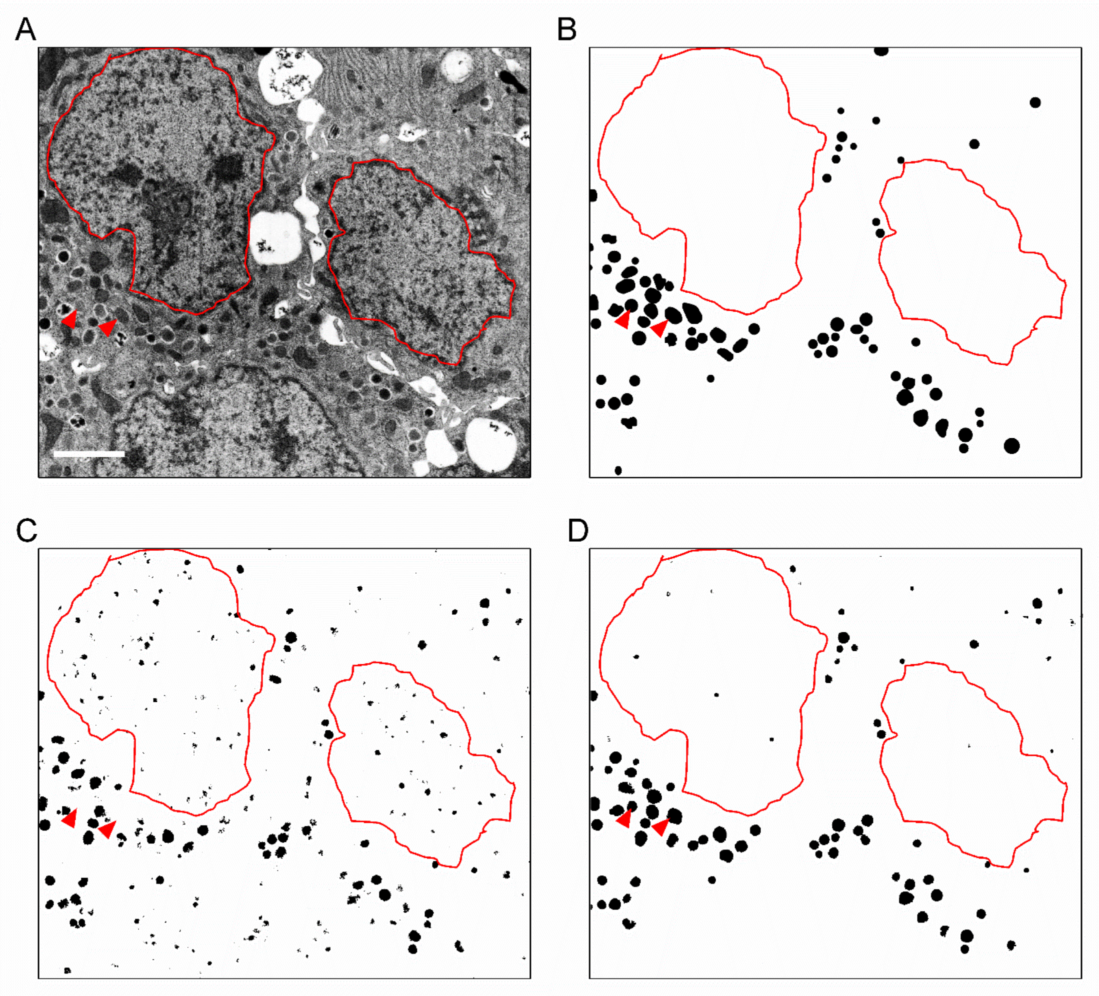

Figure S5. Comparison of performance of U-Net and MFCN in detecting insulin granules from nucleus background in WT monkey beta cells. (**A**) Three beta cells from WT rhesus monkey imaged with FIB-SEM. (**B**) Gold standard of manually segmented data. (**C**) Segmentation result of U-Net. (**D**) Segmentation result of MFCN. In (**A-D**), boundary of nucleus is labeled with red curve lines and two typical vesicles are indicated with red arrowhead. Scale bar, 2 μm.

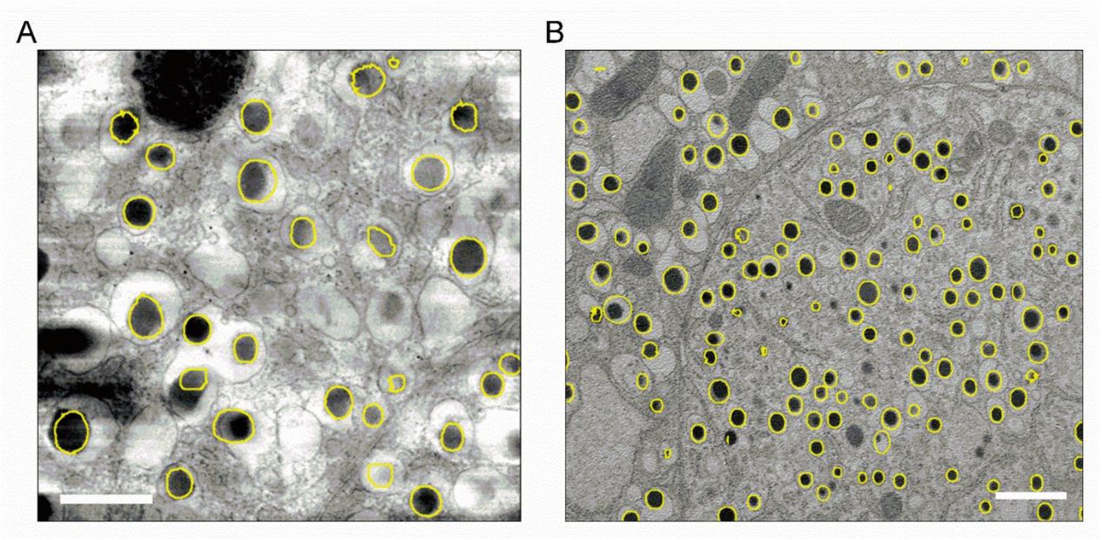

Figure S6. Segmentation of insulin granules in mouse beta cells imaged with different modes of EM by MFCN. (**A**) Our trained MFCN readily detected insulin granules in one slice of tomogram of beta cell from C57BL/6J mouse imaged with STEM tomography. Scale bar, 1 μm. (**B**) Our trained MFCN resolved insulin granules in beta cell from C57BL/6J mouse imaged with thin-slice of TEM. Scale bar, 2 μm.

Supplementary Tables

Table S1. Benchmarking of different network structures in segmenting insulin granules from both WT and MS monkeys. In some cases, we showed segmentation results (shown as mean value ± SEM) of the complete testing dataset containing both WT and MS samples. Three different structures of inception module were shown in the following figure (a-c): the number of channels of feature maps was denoted on top of the cuboids and the arrows denoted the different operations (kernel size/stride).

Inception module	Branch number	Pixel accuracy	Mean accuracy	Mean IU
b (ours)	3	0.99 ±0.0008	0.92 ±0.0050	0.89 ±0.0047
a	3	0.98 ±0.0008	0.88 ±0.0056	0.87 ±0.0050
c	3	0.98 ±0.0009	0.87 ±0.0056	0.85 ±0.0051
b	1	0.97 ±0.0011	0.84 ±0.0062	0.81 ±0.0058
b	4	0.98 ±0.0009	0.86 ±0.0056	0.84 ±0.0052
b	5	0.98 ±0.0010	0.85 ±0.0066	0.82 ±0.0057

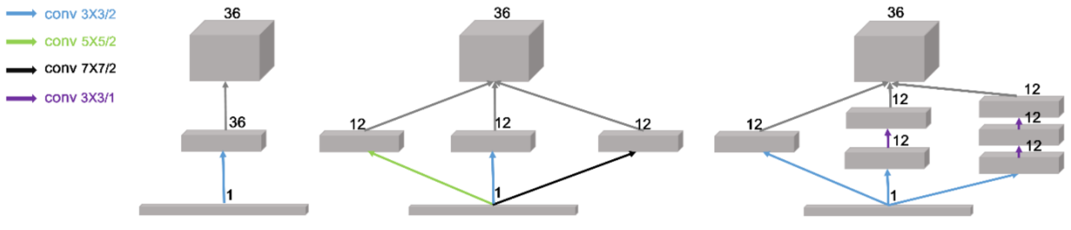

Table S2. Pixel-based accuracy and speed evaluation of various algorithms in segmenting insulin granules from both WT and MS monkeys. In some cases, we showed segmentation results (shown by the mean value ± SEM) of the complete testing dataset containing both WT and MS samples, as well as the results for only WT samples.

Method	Animal type	Pixel accuracy	Mean accuracy	Mean IU	Segmentation speed (s/image)
Random forest	WT+MS	0.96 ±0.0037	0.72 ±0.0181	0.69 ±0.0171	~ 403
CNN	WT+MS	0.97 ±0.0048	0.73 ±0.0177	0.70 ±0.0163	~ 2480
U-Net	WT+MS	0.97 ±0.0013	0.82 ±0.0087	0.75 ±0.0074	~ 3
	WT	0.97 ±0.0018	0.86 ±0.0132	0.78 ±0.0110	
MFCN	WT+MS	0.99 ±0.0010	0.92 ±0.0057	0.89 ±0.0061	~ 3
	WT	0.99 ±0.0012	0.95 ±0.0062	0.92 ±0.0055	

Table S3. Granule-based comparison of various algorithms in segmenting insulin granules from both WT and MS monkeys. In some cases, we showed segmentation results (shown as mean value ± SEM) of the complete testing dataset containing both WT and MS samples, as well as the results for only WT samples.

Method	Animal type	Precision	Recall	Miss	Error	F-measure
Random forest	WT+MS	0.51 ±0.0529	0.51 ±0.0537	0.49 ±0.0537	0.52 ±0.0383	0.51 ±0.0559
CNN	WT+MS	0.46 ±0.1003	0.50 ±0.0777	0.50 ±0.0777	0.53 ±0.0905	0.48 ±0.0871
U-Net	WT+MS	0.68 ±0.0156	0.78 ±0.0184	0.22 ±0.0184	0.38 ±0.0312	0.73 ±0.0167
	WT	0.71 ±0.0286	0.88 ±0.0264	0.12 ±0.0264	0.22 ±0.0771	0.79 ±0.0280
MFCN	WT+MS	0.87 ±0.0134	0.89 ±0.0117	0.11 ±0.0117	0.13 ±0.0243	0.88 ±0.0118
	WT	0.91 ±0.0122	0.92 ±0.0106	0.08 ±0.0106	0.12 ±0.0147	0.91 ±0.0104

References

Chen, L. C., Papandreou, G., Kokkinos,I., Murphy,K., Yuille, A.L. (2016). DeepLab: Semantic Image Segmentation with Deep Convolutional Nets, Atrous Convolution, and Fully Connected CRFs. https://arxiv.org/abs/1606.00915.

Diaz, E., Ayala, G., Diaz, M. E., Gong, L. W., and Toomre, D. (2010). Automatic Detection of Large Dense-Core Vesicles in Secretory Cells and Statistical Analysis of Their Intracellular Distribution. Ieee-Acm Transactions on Computational Biology and Bioinformatics 7, 2-11.

Jia, Y., Shelhamer,E., Donahue, J., Karayev,S., Long, J., Girshick, R., Guadarrama, S.,Darrell, T. (2014). Caffe: Convolutional Architecture for Fast Feature Embedding. https://arxiv.org/abs/1408.5093.

Long, J., Shelhamer, E., Darrell, T. (2014). Fully Convolutional Networks for Semantic Segmentation. Vol. 39(4), pp. 640-651, IEEE Transactions on Pattern Analysis and Machine Intelligence.

Merchan-Perez, A., Rodriguez, J. R., Alonso-Nanclares, L., Schertel, A., and Defelipe, J. (2009). Counting Synapses Using FIB/SEM Microscopy: A True Revolution for Ultrastructural Volume Reconstruction. Front Neuroanat 3, 18.

Ronneberger, O., Fischer, P., and Brox, T. (2015). U-Net: Convolutional Networks for Biomedical Image Segmentation. In "Medical Image Computing and Computer-Assisted Intervention - MICCAI 2015:234-241", pp. 234-241.

Smith, T. C., and Frank, E. (2016). Introducing Machine Learning Concepts with WEKA. Methods Mol Biol 1418, 353-78.

Van Valen, D. A., Kudo, T., Lane, K. M., Macklin, D. N., Quach, N. T., DeFelice, M.

M., Maayan, I., Tanouchi, Y., Ashley, E. A., and Covert, M. W. (2016). Deep Learning Automates the Quantitative Analysis of Individual Cells in Live-Cell Imaging Experiments. PLoS Comput Biol 12, e1005177.

Zhang, X., Zhang, R., Raab, S., Zheng, W., Wang, J., Liu, N., Zhu, T., Xue, L., Song, Z., Mao, J., Li, K., Zhang, H., Zhang, Y., Han, C., Ding, Y., Wang, H., Hou, N., Liu, Y., Shang, S., Li, C., Sebokova, E., Cheng, H., and Huang, P. L. (2011). Rhesus macaques develop metabolic syndrome with reversible vascular dysfunction responsive to pioglitazone. Circulation 124, 77-86.
